# Hearing Ability with Age in Northern European Women: A New Web-Based Approach to Genetic Studies

**DOI:** 10.1371/journal.pone.0035500

**Published:** 2012-04-30

**Authors:** Lisa E. Wolber, Claire J. Steves, Tim D. Spector, Frances M. K. Williams

**Affiliations:** Department of Twin Research & Genetic Epidemiology, King’s College London, London, United Kingdom; University of Ottawa, Canada

## Abstract

Age-related hearing impairment (ARHI) affects 25–40% of individuals over the age of 65. Despite the high prevalence of this complex trait, ARHI is still poorly understood. We hypothesized that variance in hearing ability with age is largely determined by genetic factors. We collected audiologic data on females of Northern European ancestry and compared different audiogram representations. A web-based speech-to-noise ratio (SNR) hearing test was compared with pure-tone thresholds to see if we could determine accurately hearing ability on people at home and the genetic contribution to each trait compared. Volunteers were recruited from the TwinsUK cohort. Hearing ability was determined using pure-tone audiometry and a web-based hearing test. Different audiogram presentations were compared for age-correlation and reflection of audiogram shape. Using structural equation modelling based on the classical twin model the heritability of ARHI, as measured by the different phenotypes, was estimated and shared variance between the web-based SNR test and pure-tone audiometry determined using bivariate modelling. Pure-tone audiometric data was collected on 1033 older females (age: 41–86). 1970 volunteers (males and females, age: 18–85) participated in the SNR. In the comparison between different ARHI phenotypes the difference between the first two principle components (PC1–PC2) best represented ARHI. The SNR test showed a sensitivity and specificity of 89% and 80%, respectively, in comparison with pure-tone audiogram data. Univariate heritability estimates ranged from 0.70 (95% CI: 0.63–0.76) for (PC1–PC2) to 0.56 (95% CI: 0.48–0.63) for PC2. The genetic correlation of PC1–PC2 and SNR was −0.67 showing that the 2 traits share variances attributed to additive genetic factors. Hearing ability showed considerable heritability in our sample. We have shown that the SNR test provides a useful surrogate marker of hearing. This will enable a much larger sample to be collected at a fraction of the cost, facilitating future genetic association studies.

## Introduction

ARHI, also referred to as presbycusis, is a complex age-related disorder affecting 25–40% of all individuals older than 65 years. Hearing loss can cause severe communication disability, leading to social isolation [1] and incapacity to work. ARHI is a common complex trait with few risk factors reliably identified. The presentation of presbycusis is diverse, showing high variance in age of onset and severity. In most cases, both ears are affected equally by sensori-neural hearing loss. Loss of hearing sensitivity with age generally starts in the higher frequencies and progresses to the lower frequencies, leading to a characteristic down-slope in the pure-tone audiogram starting at the higher frequencies and increasing in steepness.

Complex traits may be attributed to a combination of environmental and genetic factors. Several environmental factors have been implicated in the development of ARHI. Significant environmental risk factors include noise exposure (p = 1.0×10^−17^) [2], smoking (p = 0.0009) [2], alcohol consumption (OR = 0.59 95% CI: 0.43–0.80) [3], as well as cardiovascular disease (OR = 2.0, 95% CI:1.15–3.46) [4] and diabetes mellitus (p<0.05) [5].

Heritability is defined as the proportion of phenotypic variance caused by genetic factors. Heritability is a population measure and thus depends on the variance of the trait seen in the respective population. Several heritability studies have been conducted for presbycusis resulting in heritability estimates ranging from 25%–42% for strial presbycusis (atrophy of the stria vascularis, causing a flat slightly descending audiogram [6]) in families of the Framingham cohort [7] to 75% (95% CI: 0.76–0.81) for the better ear hearing threshold level (BEHL) (0.5–4 kHz) in Finnish female twins (age range: 63–76 years) [8]. Heritability was shown to decrease with increasing age and increasing environmental risk factor exposure in a cohort of male twins [9]. In general, prevalence of ARHI is higher in men, presumably due to higher occupational noise exposure, and heritability estimates for presbycusis are higher in females [7].

Several linkage, candidate gene and genome-wide association (GWA) studies have attempted to elucidate the genetic background of ARHI. Linkage studies revealed 6 loci linked to presbycusis on Chromosomes 11, 10, 14 and 18 (LOD>1.5) [10], for the *DFNA18* locus (LOD = 2.5) [11] and a locus on chromosome 8q (LOD = 4.23) [12]. Candidate gene studies focused on loci implicated in congenital deafness and reactive oxygen species removal. Associations have been suggested for the NAT2*6A polymorphism (G590A, p = 0.013) [13], polymorphisms in *GSTT1* and *GSTM1* (p = 0.035 and p = 0.027, respectively) [14], as well as for a polymorphism in *GRHL2* (p = 8.38×10^−5^) [15]. Only the association with NAT2*6A could be replicated in an independent European cohort (p = 0.013) [14]. Variable phenotype definitions, different age ranges, sample sizes and types of study populations used for hearing ability with age in various studies impairs the comparison of results and conclusions that can be reached.

Two GWA studies have been conducted for ARHI, showing suggested associations with polymorphisms in the *GRM7* gene (p = 9×10^−5^) in a European mixed gender case-control cohort (n = 1692, age range: 53–67 years) [16] and the *IQGAP2* gene (p = 3.55×10^−7^) in the Finnish Saami (n = 347, age range: 50–75 years) [17]. Both studies initially failed to reach genome-wide significance levels, which most likely reflects low sample size [18]. The association with GRM7 has been replicated in a group of 120 subjects after 23 single nucleotide polymorphisms (SNPs) were selected and tested for association [16].

This study aimed to determine genetic causal factors of differences in hearing ability with age in middle aged female twins of Northern European ancestry using two different objective measures of hearing ability: the standard pure-tone audiogram administered in controlled conditions to twins visiting the department and a web-based speech-to-noise ratio test which twins were invited by email, to perform online from home [19]. We aimed to identify the phenotype that best reflects the characteristics of hearing loss with age in particular and to establish its heritability based on the classical twin model. The novel speech-to-noise ratio (SNR) hearing test was validated against pure-tone audiometry and shared causal factors determined using bivariate structural equation modeling.

## Methods

### Pure-tone Audiogram Data Collection

Twin subjects were recruited from the TwinsUK registry at St. Thomas’ Hospital, King’s College London (www.twins.ac.uk). This twin registry comprises mainly female, adult volunteer twins recruited previously via UK media campaigns [20]. Participants were recruited from the TwinsUK registry over the age of 40. All participants underwent a detailed questionnaire on medical and environmental risk factors relevant for hearing, including auditory diseases, self reported hearing ability, hearing aid usage, occupation and noise exposure (see File S1). An otoscopic examination followed by an air-conduction pure-tone audiogram was conducted by trained personnel under standard conditions. Audiometric measures were performed using a Madsen XETA audiometer including TDH39 headphones. Audiologic equipment was calibrated on a regular basis. All research was conducted according to the ethical standards as defined by the Helsinki declaration. Ethical approval for this study was obtained from the National Research Ethics service London-Westminster (REC reference number: 07/H0802/84). Written informed consent was obtained from all participants prior to study conduction.

### Phenotype Definition

There is no gold standard method for defining ARHI and different groups have used pure tone averages (PTAs) for different frequency ranges, standardized Z-scores [21], a better ear hearing level threshold (BEHL) [8] or principal component (PC) scores calculated in a principal component analysis [12].

The PTA and BEHL are averages calculated from pure-tone thresholds of all frequencies or 0.5 kHz–4.0 kHz for the better ear, respectively. Principal component analysis is a statistical procedure based on correlations between variables. Underlying correlations between the pure-tone thresholds are determined and summarised in new variables, referred to as PCs. In case of pure-tone audiogram data, three PCs have been reported that account for size and shape of the audiogram, respectively [12]. Log transformed pure-tone thresholds (0.125–8.0 kHz) were used in this analysis. A variable combining PC1 and PC2 was generated by subtracting PC2 from PC1.

Two criteria were applied to define a good phenotypic representation for hearing loss with age: high correlation with age and the characteristic downslope of the pure-tone audiogram. As PC2 has been shown previously to capture the slope of the audiogram [22], shared variance with PC2 was determined in a linear regression on PC2, adjusted for age and twin relatedness. Each phenotype was ranked based on these two criteria. Correlation with age was determined for PTA, PC1, PC2, (PC1–PC2) and BEHL (Table 1). Mean age, mean PTA, PC scores, (PC1–PC2) scores and BEHL were compared for MZ twins, DZ twins and unpaired twins (singletons) using Kruskal-Wallis test.

**Table 1:** Demographic and phenotypic profile of study sample with pure-tone audiogram data.

zygosity	n	age range	mean age (SD)	mean PTA (SD)	mean PC1 (SD)	mean PC2 (SD)	mean PC1–PC2 (SD)	mean BEHL (SD)
**MZ**	464	44–83	62.37 (7.93)	23.2 (10.10)	−0.01 (2.02)	0.01 (1.32)	−0.02 (2.33)	19.53 (9.91)
**DZ**	528	41–86	62.14 (8.10)	23.8 (11.58)	0.07 (2.16)	−0.06 (1.23)	0.14 (2.51)	20.26 (11.25)
**singletons**	41	41–83	62.46 (10.24)	25.3 (15.63)	0.24 (2.89)	0.32 (1.31)	−0.08 (3.13)	20.52 (15.24)
**total**	1033	41–86	62.23 (8.12)	23.5 (11.13)	0.04 (2.13)	−0.01 (1.27)	0.05 (2.46)	19.93 (10.85)

The study sample was separated into monozygotic twins (MZ), dizygotic twins (DZ) and singletons. Each group was further described by its sample size (n), age-range, mean age, pure-tone average (PTA), principal component 1 (PC1), principal component 2 (PC2) as well as their difference (PC1–PC2) and better ear hearing level threshold (BEHL) values. Measurements for the mean age, pure-tone average and principle components are given as mean with standard deviation (SD).

### Speech-to-noise Ratio Test Data Collection

The SNR test measures the ability to understand words against increasing background noise. Difficulty in following a conversation in a noisy environment is one of the main symptoms of hearing loss due to advanced age. The test used in this study was originally developed for use on a telephone [23]. The SNR test was converted for web based usage by Action on Hearing loss (previously RNID). In the test, combinations of digit-triplets (i.e. 3–4–6 spoken as three-four-six) were presented at a constant sound intensity against a variable background noise [23]. The starting speech sound intensity was adapted individually by asking the subject to increase or decrease the sound intensity until a comfortable intensity was reached at which the digit triplets could be identified correctly. The initial speech sound intensity was kept constant, while the background noise increased or decreased in steps of 2 dB, depending on whether the complete triplet was understood correctly or not, respectively. The measured speech-to-noise ratio gives the ratio of speech to noise sound intensity at which half of the presentations were understood correctly.

### Validation of Speech-to-noise Ratio Test

To determine the reproducibility of the SNR test we selected 17 individuals (age range 20–83) to perform the test twice under similar conditions (same environment and same personal computer). We compared the test results using a Bland-Altman comparison [24] and Pitman’s test of difference in variance.

The results of the web based SNR test were validated against the pure-tone audiogram data. Subjects for the validation experiment (n = 351) were selected from the TwinsUK cohort if they participated both in the pure-tone audiogram and the SNR. The validation experiment included only female participants. The SNR validation cohort showed a similar age and hearing ability distribution as the pure-tone audiogram cohort (mean age: 59.8±8.5; mean PTA: 21.4±10.2; mean SNR: −10.1±2.2; all values given with±standard deviation). The correlation of the SNR test results were determined for different pure-tone audiogram presentations (PTA, BEHL, PC1,PC2 and PC1–PC2). Sensitivity and specificity of the SNR test was compared to a PTA>40 dB and a (PC1–PC2) score of 3.4 as thresholds for good hearing ability. Receiver operating curves were calculated. To determine shared causal factors between both hearing tests, bivariate structural equation modelling based on a correlated factors model was performed for PTA and SNR as well as (PC1–PC2) and SNR. For the bivariate analysis, SNR data was normalized using a cubic transformation. 

### Univariate Heritability of Different Phenotypes

Heritability estimates for hearing ability with age as a quantitative trait were calculated using structural equation modelling in Mx [25] on the basis of the classical twin model. The model assumes that phenotypic variance is determined by additive genetic (A), unique environmental (E) and common environmental (C) factors. MZ twins share all of their additive genetic factors, whereas DZ twins share on average half of their additive genetics. Common environmental factors are fully shared within MZ and DZ twin pairs, while unique environmental factors are unshared within twin pairs. In the structural equation modelling A is modelled as twice the difference between phenotypic covariance in MZ and DZ twin pairs. E is modelled as phenotypic variation not explained by shared additive genetics [1-cov(MZ) ]. As phenotypic similarity in dizygotic twins is caused by shared genetic and common environmental factors, C is calculated as the phenotypic correlation in dizygotic twins minus half the heritability. Mx compares reduced models (AE, CE and E) to the saturated ACE model using a −2 log likelihood statistic. Heritability was estimated for PTA, PC1, PC2 and BEHL and (PC1–PC2).

## Results

### Description of Study Sample with Pure-tone Audiogram Data

Pure-tone audiograms and hearing questionnaire data were collected from 1033 female participants, including 264 DZ and 232 MZ pairs and 41 singletons. The mean age was 62.23 years (± 8.12 years of standard deviation), range 41–86 years. There was no significant age-difference (p = 0.97) for MZs, DZs and singletons (Table 1). PCA was conducted and the first two principal components, PC1 and PC2, gave eigenvalues >1 and were therefore considered important components. Together, PC1 and PC2 captured 78% of the variation in the pure tone audiogram data. While the loadings for PC1 were stable across all frequencies, loadings for PC2 were high for the low frequencies (0.125–1.0 kHz) and decreased for the higher frequencies (2.0–8.0 kHz).

Pure-tone audiogram data as measured by PTA, PC1, PC2, (PC1–PC2) and BEHL did not differ significantly between MZs, DZs and singletons (PTA: p = 0.88; PC1: p = 0.86; PC2: p = 0.06; PC1–PC2: p = 0.52; BEHL: p = 0.48). PC1 values increased with raised hearing thresholds whereas PC2 values decreased with increasing downslope in the audiogram (high frequency hearing loss, increasing with steepness of slope and affection of lower frequencies). We concluded that a low PC1–PC2 value (i.e. low PC1-high PC2) would reflect good hearing ability, whereas a high PC1–PC2 value (i.e. high PC1-low PC2) would represent hearing loss with age. The highest correlation with age was determined for the combined variable of PC1–PC2 (r = 0.61) (Table 2). This combined variable also explained the highest proportion of variance in PC2 (R^2^ = 0.26) and thus resulted in the lowest rank (Table 2).

**Table 2:** Ranking of pure-tone audiogram phenotypes.

phenotype	r(age) [rank]	proportion of variance shared with PC2 [rank]
**PC1–PC2**	0.61 [1]	0.26 [1]
**PTA**	0.55 [2]	0.08 [3]
**PC1**	0.53 [3]	0.12 [2]
**BEHL**	0.49 [4]	0.08 [3]
**PC2**	−0.29 [5]	1.00 [N.A.]

Pure-tone audiogram phenotypes were ranked according to their correlation with age (Pearson’s correlation coefficient) and representation of audiogram shape (measured as proportion of variance shared with PC2). Ranks are given in square brackets.

### Speech-to-noise Ratio Test

1970 subjects responded to the web-based SNR test within the first 3 months. This first sample set included 348 MZ and 179 DZ twin pairs, as well as 916 unpaired twins. SNR scores did not differ significantly between both sexes (Kruskal-Wallis test, p = 0.09). SNR scores ranged from 6 dB −13.5 dB (Table 3).

**Table 3:** Demographic and phenotypic profile of study sample with speech-to-noise ratio data.

zygosity	n	sex (M/F)	mean age (SD)	age range	mean SNR	min SNR	max SNR
**MZ**	696	70/627	53.3 (2.08)	20–85	−10.26	−13.25	4.38
**DZ**	358	28/330	57.2 (2.17)	28–78	−10.19	−13.5	6.00
**singletons**	916	143/772	53.63 (2.18)	18–84	−13.25	−13.25	6.00
**total**	1970	241/1729	54.18 (2.14)	18–85	−10.21	−13.5	4.38

Test participants for the SNR test were described by zygosity (MZ, DZ, singletons), sample size (n), sex (male (M), female (F)) of participants, mean age with standard deviation (SD) and the age range. Mean speech-to-noise ratios (SNR) are given with minimal (min SNR) and maximal (max SNR) values.

### Validation of Speech-to-noise Ratio Test in Comparison to Pure-tone Audiometry

The results of the SNR test were reproducible with a mean difference between repeat measurements of −0.43 (95% CI: (−0.956) – 0.095). Pitman’s test for difference in variance showed no significant difference (p = 0.879).

**Figure 1:**
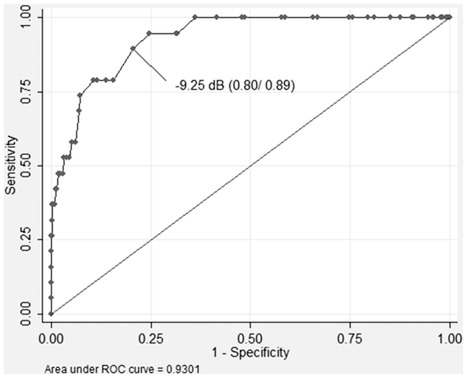
Receiver operating curve for SNR in comparison to PTA>40 dB. The receiver operating curve plots the specificity versus the sensitivity of the Speech-to-noise ratio in comparison to hearing ability measured by the pure-tone average >40 dB.

**Figure 2:**
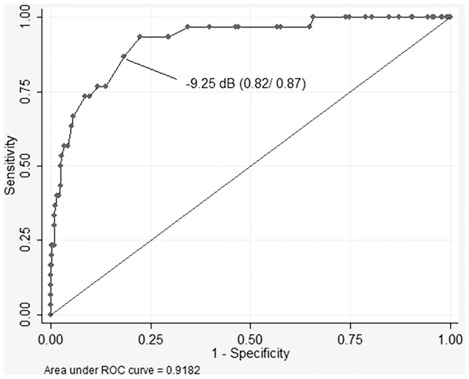
Receiver operating curve of SNR in comparison to PC1–PC2>3.4. The receiver operating curve plots the specificity versus the sensitivity of the Speech-to-noise ratio in comparison to hearing ability measured by a (PC1–PC2) >3.4.

The highest correlation with the SNR was found with PTA (r = 0.62). We decided to validate the SNR test results against PTA and PC1–PC2, which gave the best ranking in the comparison of pure-tone audiogram phenotypes. Moderate hearing impairment is defined as a PTA> 40 dB [26]. We used this criterion as the reference measurement to determine the sensitivity and specificity of the SNR test in subjects having completed both pure-tone audiogram and SNR (n = 351), although the PTA> 40 dB might not be the best criterion for hearing loss due to age. A PTA of 40 dB corresponded to a (PC1–PC2) score of 3.4. Receiver operating curves were calculated that plot 1-specficity versus sensitivity of the SNR test against a PTA threshold of 40 dB (Figure 1) and a (PC1–PC2) threshold of 3.4 (Figure 2). For a threshold of −9.25 dB SNR, a sensitivity of 89% and a specificity of 80% were obtained in comparison to the PTA (Figure 1). For a threshold of −9 dB SNR, a sensitivity of 87% and a specificity of 82% were determined when compared to (PC1–PC2) (Figure 2).

**Figure 3:**
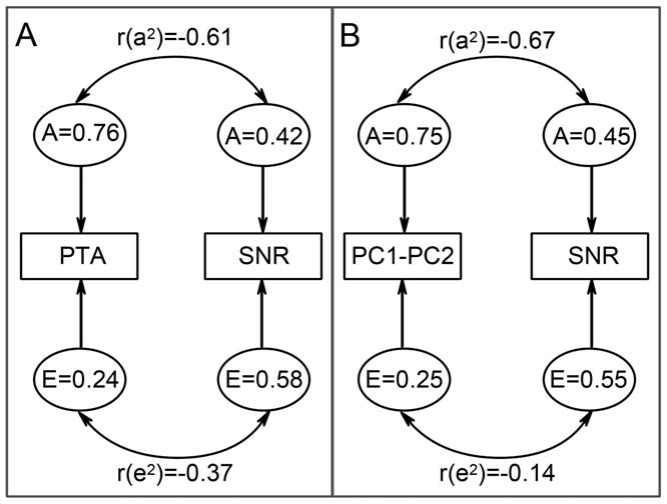
Results of bivariate variance component modelling. Graphical presentation of bivariate variance modelling results for PC1–PC2 &SNR (Panel A) and PTA & SNR (Panel B). Univariate heritability (A) and unique environmental factor (E) estimates are given separately for each trait. Correlation between these factors are given by r(a^2^) and r(e^2^), respectively.

Tests measuring the same trait might also reflect a high proportion of shared genetic factors. We investigated bivariate heritability between the two best pure-tone audiometry measures ((PC1–PC2) and PTA) and SNR results. For all bivariate analyses, the AE model gave the better model fit compared to the saturated ACE model (Table S1). Correlations between the additive genetic estimates for both tests ranged from −0.61 for age-adjusted PTA & SNR scores to −0.67 for age-adjusted (PC1–PC2) & SNR values (Figure 3, Table S1). This corresponded to 65% and 88% of phenotypic correlation due to genetic effects for PTA & SNR and PC1–PC2 & SNR, respectively.

### Univariate Heritability Study for Pure-tone Audiogram Phenotypes

Univariate structural equation modelling was used to determine the variance in phenotype explained by additive genetic factors (heritability), common environmental and unique environmental factors for different ARHI phenotype presentations. There was a high correlation with age, so age-adjusted residuals were calculated. Results of the univariate heritability study are given in Table 4. Model fit was determined using a −2 log Likelihood (−2 log L) statistic. Different nested models (AE, CE, E) were compared to the saturated ACE model based on a −2 log L difference test [27]. The Akaike information criterion (AIC) gives a measure of model fit, taking into consideration the balance of the χ^2^ statistic and the number of degrees of freedom. For each phenotype the saturated ACE model as well as nested models with better model fits are reported (Table 4). The AE model gave the best model fit for all phenotype representations. Heritability estimates ranged from 0.70 (95% CI: 0.64–0.76) for PC1–PC2 to 0.57 (95% CI: 0.49–0.63) for PC2 (Table 4).

**Table 4:** Results of univariate variance modelling for age-adjusted pure-tone audiogram phenotypes.

phenotype	model fit			model comparison				estimates % (95%CI)		
	Model	−2 log L	df	Δ −2 log L	Δ df	p-value	AIC	A	C	E
**PTA age-adjusted**	ACE	7386.954	1025	−	−	−	−	67 (52–73)	0.0(0.0–12)	33 (27–41)
	AE	7386.954	1026	0.000	1	1.000	−2.000	67(59–73)	−	33 (27–41)
**PC1 age-adjusted**	ACE	4006.850	1025	−	−	−	−	59 (35–69)	3 (0.0–23)	38 (31–46)
	AE	4006.919	1026	0.069	1	0.792	−1.931	63 (55–69)	−	37 (31–45)
**PC2 age-adjusted**	ACE	3235.682	1025	−	−	−	−	40 (14–62)	16 (0.0–37)	45 (37–53)
	AE	3237.390	1026	1.709	1	0.191	−0.291	57 (49–63)	−	43 (37–51)
**(PC1–PC2) age-adjusted**	ACE	4167.301	1025	−	−	−	−	70 (58–76)	0.0 (0.0–11)	30 (24–36)
	AE	4167.301	1026	0.000	1	1.000	−2.000	70 (64–76)	−	30(24–36)
**BEHL age-adjusted**	ACE	7421.538	1025	−	−	−	−	65 (54–72)	0.0 (0.0–8.0)	35 (28–42)
	AE	7421.538	1026	0.000	1	1.000	−2.000	65 (58–72)	−	35 (28–42)

Three nested models were compared to the saturated ACE model, taking into account different causal factors: AE (additive genetics and unshared environmental factors), CE (shared and unshared environmental factors) and E (unshared environmental factors). For each phenotype the saturated model and nested models with a better model fit (minus 2 log likelihood(−2 logL), degrees of freedom (df)) are shown. Model comparison is only given for nested models as they are compared to the full (ACE) model. Estimated variances explained by the specific causal factors (A =  additive genetics, C =  shared environment and E =  unshared environment) are given with 95% confidence intervals for each model.

## Discussion

For this study, we collected pure-tone audiogram data and SNR data of 1033 and 1970 mainly female participants, respectively. (PC1–PC2) was suggested as new phenotype definition for hearing ability with age, which showed a high correlation with age and best captured high frequency hearing loss. We have shown that the SNR is a valid tool when compared with the standard test and will allow us to increase our sample size for future GWAs on hearing ability with age as a quantitative trait. The moderate univariate heritability determined for pure-tone audiogram phenotypes suggests causal genetic factors involved in the etiology of hearing ability with age.

The TwinsUK cohort has been shown to be widely representative of the general singleton North European population [28] and their involvement in numerous genome wide association study meta-analyses attests to their comparability with other populations [29].

For the pure-tone audiogram representations, the values of the better ear were chosen for analysis, as it is considered to be less representative of environmental factors affecting hearing ability. Exposure to such factors would not represent natural age-related hearing and therefore bias the search for an ageing phenotype. We decided not to adjust for noise exposure in this study, due to minimal self reported exposure in the presented cohort (less than 8% of participants reported to have been exposed to occupational noise for more than one year). In addition, noise exposure was not significantly associated with hearing ability (p = 0.92) in our cohort.

Several phenotypes have been used to represent hearing ability with age in previous studies, however, no gold standard could be decided on. Moderate hearing impairment (PTA>40 dB), as defined by the WHO [26] is a very stringent criteria. In addition, averaging hearing ability over all frequencies (i.e. PTA) might not be suitable to measure hearing ability with age as it neglects the characteristic downslope of the audiogram. We therefore compared different phenotypes as measured by pure-tone audiometry including PTAs, BEHL, scores for PC1 and PC2. Two criteria important for hearing impairment with age were defined: the correlation with age (for an age-related trait) and capture of the characteristic down-slope seen for high frequency hearing loss, the most common variant of ARHI. As PC1 and PC2 alone captured only fractions of the variance in pure-tone audiograms, we decided to combine both variables by taking their difference (PC1–PC2). In the ranking according to the above criteria, (PC1–PC2) gave the best rank. We therefore propose (PC1–PC2) as a new phenotype to measure ARHI from pure-tone audiogram data. We acknowledge that principal component loadings are dependent on the study population and more complicated to adapt than PTAs but their advantage is that they capture magnitude and shape of the audiogram, whereas average variables (PTA, BEHL) mainly represent the magnitude alone. Nevertheless, this problem could be targeted by deciding on equal PC loadings in replication studies or meta analyses.

In this study, we validated a measure of hearing ability obtained via the web, the SNR test. This test has previously been validated for telephone usage [23] producing a sensitivity of 79% and specificity of 100% of the BEHL as measured by pure-tone audiometry versus the telephone based SNR [23]. A new method should always be validated against the existing standard measure: we therefore determined the sensitivity and specificity of the web based SNR against the PTA and (PC1–PC2) as obtained by the pure-tone audiogram. Sensitivity and specificity was high in comparison to the PTA (89% and 80%, respectively), but decreased slightly when the SNR was validated against (PC1–PC2) (87% and 82%, respectively). This could be expected considering that PC1 and PC2 together account for 78% of the variances in pure-tone thresholds while the PTA reflects 100%.

These sensitivity and specificity values might be too low for a diagnostic test, but appropriate for a screening test, as used here. Word recognition and hearing sensitivity at different frequencies represent different aspects of ARHI and might be explained by different causal factors. To reduce the effect of cognitive ability on word recognition digit combinations were used as speech material rather than complete sentences. We hypothesized that variances in both measurements could be explained by shared causal genetic factors and our bivariate heritability results underline this notion. Genetic correlations between pure-tone audiogram measurements (PTA and (PC1-PC2)) and SNR determined in the bivariate analyses ranged from r =  −0.61 to r = −0.67. According to the sensitivity and specificity as well as the high bivariate heritability correlations determined, we conclude that the SNR represents a satisfactory surrogate to the pure-tone audiogram and that both tests could serve as complements to each other.

The heritability estimates from the univariate analysis ranged from 70% (95% CI: 0.64–0.76) for (PC1–PC2) to 57% (95% CI: 0.49–0.63) for PC2. This is comparable to a heritability of 75% for pure-tone thresholds reported for female Finnish twins [8] and a heritability of 66.3% for PC1 achieved in a population of singletons [12]. Our twin sample was composed exclusively of females with relatively minor exposure to environmental risk factors, high occupational status and a broader age range and lower mean age than previously conducted heritability studies on ARHI. Therefore, a lower phenotypic variance could be expected for this population due to decreased heterogeneity.

The moderate to high heritability estimates confirm the involvement of genetic factors in the etiology of ARHI as well as the reliability of the phenotype measure.

We accept that there were limitations to our data collection. Conductive hearing loss, commonly diagnosed as an air-bone gap of >20 dB, could not be fully excluded, due to a lack of bone conduction measurements. However, auditory diseases leading to conductive hearing loss (i.e. Otosclerosis, cholesteatoma and chronic otitis media) were covered in the questionnaire and subjects were excluded upon these pathologies. In addition, hearing loss generally refers to a longitudinal process, whereas the data we presented here was cross-sectional. The percentage of subjects with moderate hearing loss as defined by the WHO (PTA>40 dB) [26] was relatively low for the TwinsUK cohort. This might be explained by better hearing ability than average for this cohort. Considering that ARHI affects predominantly the higher frequencies, elderly subjects with a PTA<40 dB might still be affected by ARHI. For the validation of the SNR test, the number of subjects having performed both tests was limited (n = 351, only females) compared to the overall sample size. However, the audiometric profile of the SNR validation cohort resembled that of the complete pure-tone audiogram and SNR cohort. We also acknowledge that the web-based test attracted slightly younger subjects and was not limited to female participants like the pure-tone audiogram. We will address this by a correction for age and gender in the future analysis of this data set.

## Supporting Information

File S1 **Copy of hearing questionnaire.** The Hearing questionnaire was developed to test for self reported hearing loss and exposure to environmental risk factors for hearing ability.
(DOCX)

Table S1 **Results of bivariate variance modelling for PTA & SNR and PC1–PC2 & SNR.** Results of bivariate structural equation modelling are given for unadjusted and age-adjusted pure-tone audiogram phenotypes. The nested AE models were compared to the saturated ACE model. Model fit is given as minus 2 log likelihood (−2 log L), degrees of freedom (df) and difference in −2 log L and df between the nested and saturated model (Δ −2 log L, Δ df) are shown. Estimated variances explained by the specific causal factors (A =  additive genetics, C =  shared environment and E =  unshared environment) are given with 95% confidence intervals for each model. For each model, univariate estimates for each phenotype separately and the correlation (r(a^2^), r(e^2^) and r(c^2^)) between these estimates are reported.
(DOCX)
