# Supplementary material for: Hearing Ability with Age in Northern European Women: A New Web-Based Approach to Genetic Studies
Source: PLoS One. 2012 Apr 30;7(4):e35500. doi: 10.1371/journal.pone.0035500 (PMC3340381; doi:10.1371/journal.pone.0035500)
Supplement: File S1 — Copy of hearing questionnaire. The Hearing questionnaire was developed to test for self reported hearing loss and exposure to environmental risk factors for hearing ability. (DOCX) [file pone.0035500.s001.docx]

# File S1.Copy of hearing questionnaire

**Learning, memory and hearing questionnaire**

**Healthy Ageing Twin Study**

**Your hearing:**

| 2.1 **Do you have any difficulty**  **with your hearing?** | No | Yes | | | | | | Not known |
| --- | --- | --- | --- | --- | --- | --- | --- | --- |
| 2.2 **Have you ever had an ear disease?** | No | **Yes** Acute ear inflammation **in childhood** (>3 times) with pain, discharge. | | | | | | Not known |
| (> more than) |  | **Yes** Acute ear inflammation **as an adult** (>3 times) with pain and discharge | | | | | | Not known |
|  |  | **Yes** Chronic ear inflammation with   a feeling of deafened ear, discharge from ear (lasting >3 months) | | | | | | Not known |
| 2.3 **Or an ear operation?** | No | **Yes** Plastic tube   through ear drum  **Yes** Other  ear drum operation  **Yes** Ossicle operation  **Yes** Infection in bones  behind ear (Mastoiditis)  **Yes** Cholesteatoma of  middle ear  **Yes** Otosclerosis  (broken ossicle) | | R  R  R  R  R  R | L  L  L  L  L  L | |  | Not known    Not known    Not known  Not known  Not known  Not known |
| 2.4 **Have you ever experienced an explosion** **or gunfire** which caused immediate hearing loss or tinnitus? | No | Yes | | | | | | Not known |
| 2.5 **Have you been frequently exposed to loud noise in your leisure time without using protection.** (Frequently would be more than once a month over several years) | | | | | | | | |
|  | No | Yes – loud music  Yes – noisy handiwork / power tools  Yes – gunshots | | | | Not known | | |
| 2.6 **Do you wear a hearing aid?** | No | Yes | | | | Not known | | |
| 3.1 **What best describes your main occupation throughout most of your life?**  Professional or managerial  Non-manual or clerical  Manual  Housewife  Student  None | | | 3.2 **Have you ever worked in a place that was so noisy you had to shout to be heard**?  No, never  Yes, for less than 1 year  Yes, for 1-5 years  Yes, for more than 5 years | | | | | |
