# Supplementary material for: Hearing Ability with Age in Northern European Women: A New Web-Based Approach to Genetic Studies
Source: PLoS One. 2012 Apr 30;7(4):e35500. doi: 10.1371/journal.pone.0035500 (PMC3340381; doi:10.1371/journal.pone.0035500)
Supplement: Table S1 — Results of bivariate variance modelling for PTA & SNR and PC1–PC2 & SNR. Results of bivariate structural equation modelling are given for unadjusted and age-adjusted pure-tone audiogram phenotypes. The nested AE models were compared to the saturated ACE model. Model fit is given as minus 2 log likelihood (−2 log L), degrees of freedom (df) and difference in −2 log L and df between the nested and saturated model (Δ −2 log L, Δ df) are shown. Estimated variances explained by the specific causal factors (A = additive genetics, C = shared environment and E = unshared environment) are given with 95% confidence intervals for each model. For each model, univariate estimates for each phenotype separately and the correlation (r(a2), r(e2) and r(c2)) between these estimates are reported. (DOCX) [file pone.0035500.s002.docx]

**Table S1. Results of bivariate variance modelling for PTA & SNR and PC1-PC2 & SNR**

| **Phenotype** |  | **Model fit** | |  | **Model comparison** |  |  |  | **univariate Estimates % (95% CI)** |  |  | **correlation between estimates (95% CI)** |  | |  |
| --- | --- | --- | --- | --- | --- | --- | --- | --- | --- | --- | --- | --- | --- | --- | --- |
|  | **Model** | | **-2 log L** | **df** | **∆ -2 log L** | **∆ df** | **p-value** | **AIC** | **A** | **C** | **E** | **r(a^2^)** | | **r(c^2^)** | **r(e^2^)** |
| **(PC1-PC2) & SNR** | ACE | | 2917.957 | 691 | - | - | - | - | 58 (27-87) | 25 (0-55) | 16 (11-24) | -1.00 (-1.00--0.63) | | 1.00 (-1.00-1.00) | -0.10(-0.35-0.13) |
|  |  | |  |  | - | - | - | - | 53 (17-68) | 0 (0- 31) | 47 (32-66) |  | |  |  |
|  | AE | | 2921.117 | 694 | 3.160 | 3 | 0.368 | -2.840 | 84 (76-89) | - | 16 (11-24) | -0.77 (-0.94--0.63) | | - | -0.13 (-0.38-0.12) |
|  |  | |  |  |  |  |  |  | 54 (32-69) | - | 46 (31-68) |  | |  |  |
| **(PC1-PC2) & SNR**  **age-adjusted** | ACE | | 2804.185 | 686 | - | - | - | - | 64 (32-82) | 11(0-39) | 25 (17-39) | -1.00 (-1.00--0.53) | | 1.00(-1.00- 1.00) | -0.12(-0.38-0.14) |
|  |  | |  |  | - | - | - | - | 37 (10-60) | 6 (0-27) | 57 (38-80) |  | |  |  |
|  | AE | | 2806.276 | 689 | 2.091 | 3 | 0.554 | -3.909 | 75 (62-84) | - | 25 (16-38) | -0.67 (-0.93--0.46) | | - | -0.14(-0.41-0.12) |
|  |  | |  |  | - | - | - | - | 45(18-63) | - | 55 (37-83) |  | |  |  |
| **PTA & SNR** | ACE | | 2320.677 | 691 | - | - | - | - | 49(18-81) | 32 (1-60) | 19(13-29) | -1.00(-1.00--0.62) | | 1.00(-1.00-1.00) | -0.31(-0.53--0.09) |
|  |  | |  |  |  |  |  |  | 52(12-68) | 0.1 (0-32) | 48(32-70) |  | |  |  |
|  | AE | | 2326.039 | 694 | 5.362 | 3 | 0.147 | -0.638 | 81(71-87) | - | 19(13-29) | -0.73 (-0.89--0.58) | | - | -0.35(-0.56--0.10) |
|  |  | |  |  |  |  |  |  | 53 (29-68) | - | 47 (32-71) |  | |  |  |
| **PTA & SNR**  **age-adjusted** | ACE | | 2714.569 | 686 | - | - | - | - | 58(24-81) | 19 (0-48) | 24(15-38) | -1.00(-1.00--0.54) | | 1.00(-1.00-1.00) | -0.34(-0.57--0.09) |
|  |  | |  |  |  |  |  |  | 35(7-59) | 5 (0-27) | 60(40-85) |  | |  |  |
|  | AE | | 2718.389 | 689 | 3.820 | 3 | 0.282 | -2.180 | 76 (62-84) | - | 24(16-38) | -0.61 (-0.86--0.38) | | - | -0.37 (-0.60--0.11) |
|  |  | |  |  |  |  |  |  | 42 (12-62) | - | 58 (38-88) |  | |  |  |

# Supporting Material 2

## Copy of hearing questionnaire

**Learning, memory and hearing questionnaire**

**Healthy Ageing Twin Study**

**Your hearing:**

| 2.1 **Do you have any difficulty**  **with your hearing?** | No | Yes | | | | | | Not known |
| --- | --- | --- | --- | --- | --- | --- | --- | --- |
| 2.2 **Have you ever had an ear disease?** | No | **Yes** Acute ear inflammation **in childhood** (>3 times) with pain, discharge. | | | | | | Not known |
| (> more than) |  | **Yes** Acute ear inflammation **as an adult** (>3 times) with pain and discharge | | | | | | Not known |
|  |  | **Yes** Chronic ear inflammation with   a feeling of deafened ear, discharge from ear (lasting >3 months) | | | | | | Not known |
| 2.3 **Or an ear operation?** | No | **Yes** Plastic tube   through ear drum  **Yes** Other  ear drum operation  **Yes** Ossicle operation  **Yes** Infection in bones  behind ear (Mastoiditis)  **Yes** Cholesteatoma of  middle ear  **Yes** Otosclerosis  (broken ossicle) | | R  R  R  R  R  R | L  L  L  L  L  L | |  | Not known    Not known    Not known  Not known  Not known  Not known |
| 2.4 **Have you ever experienced an explosion** **or gunfire** which caused immediate hearing loss or tinnitus? | No | Yes | | | | | | Not known |
| 2.5 **Have you been frequently exposed to loud noise in your leisure time without using protection.** (Frequently would be more than once a month over several years) | | | | | | | | |
|  | No | Yes – loud music  Yes – noisy handiwork / power tools  Yes – gunshots | | | | Not known | | |
| 2.6 **Do you wear a hearing aid?** | No | Yes | | | | Not known | | |
| 3.1 **What best describes your main occupation throughout most of your life?**  Professional or managerial  Non-manual or clerical  Manual  Housewife  Student  None | | | 3.2 **Have you ever worked in a place that was so noisy you had to shout to be heard**?  No, never  Yes, for less than 1 year  Yes, for 1-5 years  Yes, for more than 5 years | | | | | |
